# In-vitro evaluation of shear bond strength and bond-failure of isoamyl cyanoacrylate-resin adhesive ratios for bonding 3D-Printed denture bases and teeth

**DOI:** 10.1016/j.jobcr.2025.11.017

**Published:** 2025-12-01

**Authors:** Divyansh Sinha, Ram Kiran, Suresh Venugopalan

**Affiliations:** Department of Prosthodontics, Saveetha Dental College, Saveetha Institute of Medical and Technical Sciences, Chennai, India

**Keywords:** Cyanoacrylate, 3D-printed dentures, Shear bond strength, Isoamyl cyanoacrylate, Additive manufacturing, Denture base–tooth bonding, Failure mode analysis

## Abstract

**Statement of problem:**

Shear bond strength between 3D-printed denture bases and teeth is inferior compared to conventional and milled dentures.

**Purpose:**

The success of 3D-printed complete dentures depends on reliable bonding between the denture base and teeth. This study evaluated shear bond strength and modes of bond failure of various isoamyl cyanoacrylate (ICA) and 3D printing resin formulations in different ratios.

**Materials and methods:**

Adhesive formulations of standardized ICA-to-resin ratios were prepared. Fifty samples were randomized into five groups: 0 % (control), 25 %, 50 %, 75 %, and 100 % ICA. Specimens were fabricated per ISO/TS 19736 standards using 3D-printed denture base and tooth resins. After bonding and polymerization, samples underwent shear bond strength testing. Failure modes were examined under stereomicroscope and classified as adhesive, cohesive, or mixed. Data were analyzed using one-way ANOVA with Tukey's post hoc test.

**Results:**

The 50 % ICA group showed the highest mean shear bond strength (3.738 ± 0.063 MPa), significantly greater than all others (P = 0.000). The control group exhibited the lowest strength (1.602 ± 0.140 MPa). Bond failure analysis showed predominantly adhesive failures in the control, whereas the 50 % ICA group had the greatest proportion of cohesive and mixed failures, indicating improved interfacial bonding and internal strength.

**Conclusions:**

A 50:50 ratio of isoamyl cyanoacrylate and light-cure 3D printing resin provides optimal bond strength and favorable failure patterns for bonding 3D-printed denture bases and teeth suggesting that a hybrid adhesive approach offers a promising solution to enhance the durability of 3D-printed dentures.

## 1 Introduction

The integrity of the bond between denture bases and denture teeth is critical for the clinical success and longevity of complete dentures. Traditional denture base materials, such as heat-cured polymethyl methacrylate (PMMA), have been widely used due to their favorable mechanical properties, ease of processing, and proven biocompatibility.^1^^,^^2^ The denture base and teeth are heat polymerized as a single unit, hence, they demonstrate strong chemical bonding. The advent of digital technology has profoundly revolutionized the field of prosthodontics.^3^ Among these advances, additive manufacturing by 3D printing has gained prominence for producing customized denture bases and teeth which aim to overcome many of the limitations inherent to conventional denture fabrication techniques, such as polymerization shrinkage, human error in wax-up and processing, and labor-intensive laboratory steps.^4^^,^^5^ These dentures allow for a more accurate fit, reduced material wastage, and faster production, with reduced clinical appointments and laboratory time.^6^^,^^7^ This is advantageous to both the clinician as well as the dental laboratory. Despite the advances in digital workflows, the weak interface between separately printed denture bases and teeth remains a significant clinical concern. Failures at this junction can compromise the functional longevity of complete dentures, leading to debonding, frequent repairs, and reduced patient satisfaction. These failures not only affect prosthesis durability but also impose additional financial and psychological burdens on patients, particularly elderly individuals who rely heavily on removable prostheses for mastication, speech, and aesthetics.^8^ Enhancing the adhesive strength at the denture base-tooth interface is therefore critical for the long-term clinical success of 3D-printed dentures. To address this issue, researchers and manufacturers have investigated various methods to enhance the bond strength between 3D-printed denture components. These include surface treatments such as air abrasion, application of bonding agents, and the use of dual-cure or light-cure adhesives.^10^, ^11^, ^12^, ^13^, ^9^ However, these methods often present limitations related to clinical applicability or lack of long-term durability. An adhesive system that provides reliable bonding could reduce fracture and repair rates, extend the service life of prostheses, and ultimately improve patient comfort and confidence in wearing dentures. Traditionally, the use of light-cure 3D printing resins for denture base and tooth fabrication introduces a new set of chemical and mechanical challenges. These resins are often based on urethane dimethacrylate (UDMA), bisphenol A-glycidyl methacrylate (Bis-GMA), or similar dimethacrylate monomers that polymerize upon exposure to visible light.^11^ While these materials offer advantages such as low shrinkage, color stability, and high surface hardness, their cross-linked nature limits post-polymerization bonding capacity. Achieving a strong bond between two cured 3D-printed components is especially difficult, as interpenetration or chemical diffusion is minimal. Therefore, the incorporation of a secondary bonding system is paramount, such as a cyanoacrylate-based adhesive, which may help bridge the interface by providing mechanical adhesion as well as potential chemical linkage.^10^

Cyanoacrylate adhesives, commonly referred to as superglues, are widely known for their rapid polymerization upon exposure to moisture and strong adhesive properties due to which they are being extensively used in industrial, medical, and dental applications due to their ability to form strong bonds between various substrates.^14^^,^^15^ Among the different types of cyanoacrylates available, short-chain variants such as ethyl cyanoacrylate are commonly used for industrial adhesion, whereas longer-chain formulations like n-butyl, 2-octyl and isoamyl cyanoacrylate have been preferred in medical applications such as tissue adhesives and wound closure due to their improved flexibility, reduced cytotoxicity, and enhanced mechanical performance.^16^ In dentistry, cyanoacrylate adhesives have been explored for applications in denture repair, orthodontics, and temporary bonding of prosthetic components. Isoamyl cyanoacrylate (ICA), a longer-chain cyanoacrylate, has shown potential in medical and dental applications due to its flexibility and improved bond strength compared to conventional cyanoacrylates.^14^^,^^17^^,^^18^ While cyanoacrylates have been widely investigated for various adhesive applications, their specific efficacy in bonding 3D-printed denture bases and teeth remains underexplored.

The ideal adhesive formulation should provide a balance between mechanical strength, biocompatibility, and resistance to degradation under oral conditions. Previous studies have demonstrated that modifying the adhesive composition can significantly impact the bond strength and failure resistance of dental prostheses.^6^^,^^8^^,^^11^^,^^19^^,^^20^ However, no study to date has systematically evaluated the effect of using ICA and light-cure printing resin in different compositional ratios for bonding 3D-printed denture components. Therefore, an optimized ratio of ICA and 3D-printing resin may be a reliable adhesive strategy tailored to enhance adhesion between denture bases and teeth, reducing debonding failures and the need for frequent denture repairs, improving the clinical durability and patient satisfaction for 3D-printed prostheses. In this context, the present study explores the feasibility of using a hybrid adhesive system composed of ICA and light-cure 3D printing resin in varying ratios. By systematically analyzing the bond strength of various mixtures, we can provide valuable insights into improving adhesive formulations for clinical applications. This study aims to evaluate the shear bond strength of different ratios of ICA and 3D-printing dental resin to identify the optimal composition for enhanced adhesion of 3D-printed denture components. Given this gap, it is essential to determine whether altering the ICA–resin ratio produces a measurable difference in shear bond strength. The null hypothesis was there will be no difference in the shear bond strength values between 3D-printed denture base and tooth samples, when bonded using different compositional combinations of 3D-printing resin and ICA.

## 2 Materials and methods

**Fig. 1:**
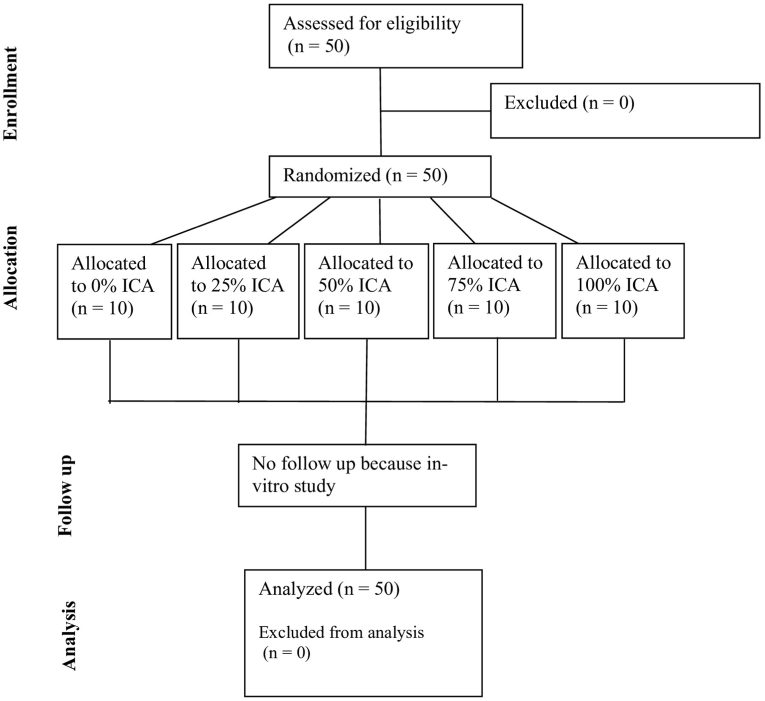
Schematic flowchart of the study workflow showing the study methodology, including enrollment, randomization and allocation, follow up and testing procedures.

**Fig. 2:**
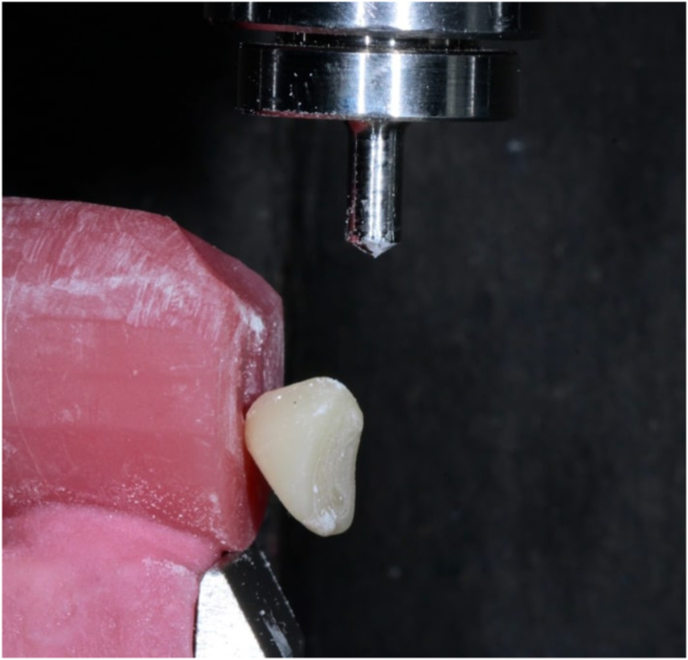
Shear bond strength analysis setup using the Instron 3000 Universal Testing Machine. The sample held with the positioning jig shows fracture after applying a vertical downward force using the shear testing rod.

The study design involved five groups based on the ratio by weight between photocurable printing resin and cyanoacrylate adhesive, transitioning from a resin-dominant adhesive to a cyanoacrylate-dominant adhesive: Group 1, 100 % 3D-printing dental resin (0 % ICA), Group 2, 75 % 3D-printing resin +25 % ICA (25 % ICA), Group 3, 50 % 3D-printing resin +50 % ICA (50 % ICA), Group 4, 25 % 3D-printing resin +75 % ICA (75 % ICA) and Group 5, 100 % ICA (100 % ICA). The 0 % ICA group, representing conventional light-cure resin adhesive, served as a baseline control to evaluate the relative enhancement achieved by ICA addition. 5 ml adhesives were prepared of each formulative ratio. The required resin and adhesive volumes were calculated to ensure accuracy and repeatability across samples. A precision micropipette was used to dispense measured quantities of the adhesive and resin into graduated cylinders accurately. Mixing was performed at 23 ± 1 °C and relative humidity of 40–50 % using a sterile glass rod without introducing air bubbles under low humidity-controlled conditions to prevent premature polymerization. The solutions were gently stirred for 10 s until homogeneity was achieved. The cylinders were immediately sealed to avoid premature moisture-induced polymerization and were used for testing within 5 min of adhesive preparation. The study used 3D-printed samples of denture bases and artificial teeth created from photo-curable acrylic resins NextDent Denture 3D for bases and NextDent C&B for teeth (Vertex-Dental B.V., Netherlands). Sample size was calculated to be 50 (10 per group) using GPower 3.0 software according to a pilot study performed, with considering the probability of type 1 error α = 0.05, study power (1-β) of 0.90 and an effect size of 1.5773. Sample preparation and testing methodology was standardized according to the ISO/TS 19736 standard for shear bond strength and failure mode analysis. Fifty cylindrical bases of height 20 mm and diameter 25 mm were used to simulate the denture base material with a round depression of 1 mm of diameter 7 mm were designed using TinkerCad (AutoDesk, USA) software of the above measurements. Using the denture base design STL, the denture teeth samples were designed. The design of each tooth specimen was of a maxillary right central incisor of height 10 mm from the generic library of ExoCAD software (ExoCAD GmbH, Germany), vertically placed on the denture base extending 1 mm above the neck of the tooth. The samples were printed using the NextDent 5100 3D printer with layer thickness of 50 μm, at 0° build orientation. Subsequent post-curing was performed for 20 min under 405 nm LED light (intensity 50 mW/cm^2^) in the LC-3D Print Box according to manufacturer instructions (Vertex Dental B.V., Netherlands). For clarity, a flowchart illustrating the group allocation, randomization and shear bond strength testing procedures has been included (Fig. 1), providing a visual summary of the study workflow. The samples were numbered and were randomly allotted to either one of the groups using Random Allocation software 2.0, designed by Mahmood Saghaei.^21^ No blinding was used during data collection due to the visible differences in material behavior; however, blinding was applied during outcome assessment, as the investigator performing shear bond measurements and failure mode analysis was blinded to group allocation to minimize bias during outcome assessment. The cylinders with the socket simulated the denture base into which the denture teeth can be placed. Each sample was coated with the adhesive according to the manufacturer's guidelines, with a thin single layer applied to the bonding surface of the denture base using a microbrush to avoid excess adhesive buildup. The artificial tooth was then positioned with firm digital pressure for 2 min to eliminate air pockets and ensure intimate contact. Any excess was cleaned with a gauze swab and the samples were cured in a light cure chamber according to manufacture recommendations for 3D Printed resin and were immediately tested. No surface treatment or pre-conditioning was performed on the bonding surfaces to ensure that the observed differences in bond strength were solely attributable to variations in adhesive composition rather than surface modification effects. Shear bond strength testing was performed using a universal testing machine (Instron E3000, USA) which applied a perpendicular force with a crosshead speed to 1 mm/s to the bonded surface until failure of the sample occurred as seen in Fig. 2. The maximum force at the point of bond failure was recorded for each sample in Newton (N), which was divided by the bonded surface area (mm^2^) to yield shear bond strength (MPa). The universal testing machine (Instron E3000, USA) was calibrated before each test session according to ISO 7500–1:2018 standards to ensure accuracy of load measurements. All bond strength tests were conducted at room temperature (23 ± 2 °C) and 50 % relative humidity. The type of bond failure was classified into adhesive, cohesive and mixed failures. Adhesive failure occurred between the interfaces of 3D-printed artificial acrylic tooth and 3D-printed denture base samples. Cohesive failure occurred within either of 3D-printed artificial acrylic tooth or 3-D printed denture base samples. Mixed failure was a combination of both the failures. The fracture surfaces were inspected using a stereomicroscope (Leica M205C, Leica Microsystems, Switzerland) to determine the mechanism of bond failure. No samples were excluded; all 50 specimens were successfully tested and included in analysis.

### 2.1 Statistical analysis

Data were analyzed using IBM SPSS Statistics 23.0 software (IBM Corp., Armonk, Chicago, USA). Shapiro-Wilk's test of normality was individually performed for each group which showed that all datasets followed normal Gaussian distribution. Hence, one-way fixed effects Analysis of Variance (ANOVA) was conducted to compare mean bond strengths between groups, followed by Tukey's post-hoc multiple comparisons analysis to determine specific differences among the shear bond strengths. Prior to performing one-way ANOVA, the assumption of homogeneity of variances was assessed using Levene's test. P-value less than 0.05 was considered statistically significant. Spearman rank-order correlation test was additionally performed to assess the association between shear bond strength and failure modes.

## 3 Results

**Fig. 3:**
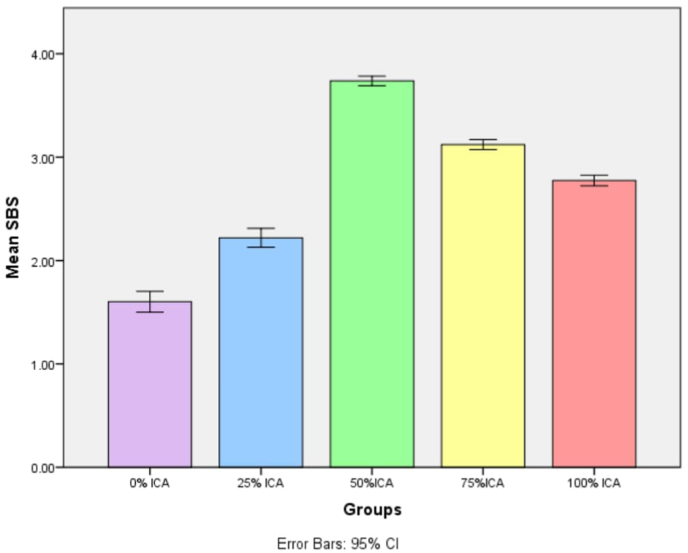
Mean shear bond strength values (95 % CI error bars) for the different adhesive formulation groups. The 50 % ICA blend demonstrated the highest mean bond strength, significantly higher than all other groups (p < 0.001).

**Fig. 4:**
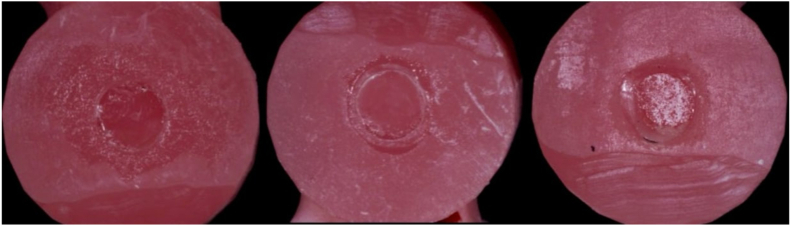
Type of bond failure: Cohesive (left), adhesive (centre) and mixed (right), measured under a stereomicroscope.

**Fig. 5:**
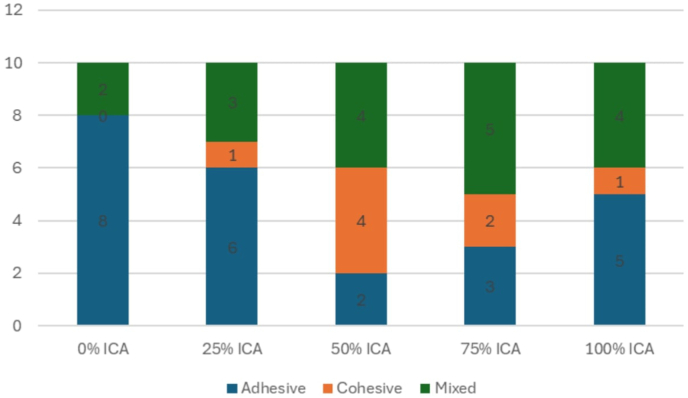
Comparative evaluation of the type of bond failure among groups.

The mean shear bond strength values for the five groups with varying ratios of ICA to light-cure 3D printing resin are presented in Table 1. The mean shear bond strengths (mean ± SD, 95 % CI) were 1.60 ± 0.14 MPa (1.50–1.70) for the 0 % ICA group, 2.22 ± 0.13 MPa (2.13–2.31) for the 25 % ICA group, 3.74 ± 0.06 MPa (3.69–3.78) for the 50 % ICA group, 3.12 ± 0.07 MPa (3.07–3.17) for the 75 % ICA group, and 2.77 ± 0.07 MPa (2.72–2.83) for the 100 % ICA group as shown in Fig. 3. Levene's test indicated that the variances across the five groups were approximately equal (Levene's statistic = 2.577, df = 4, 45, P = 0.050), confirming the homogeneity of variance assumption for ANOVA. Although variance equality was confirmed, the 75 % ICA group demonstrated a broader distribution of values, likely attributable to heterogeneity in polymerization kinetics and mixing uniformity at higher ICA proportions. A one-way analysis of variance (ANOVA) with Tukey's post hoc analysis revealed a statistically significant difference in shear bond strength among the groups (*P* = 0.000) as shown in Table 2. Tukey's post hoc test confirmed that the 50 % ICA group was significantly stronger than all other groups (*P* = 0.000). The 25 %, 75 %, and 100 % ICA groups also demonstrated significantly higher bond strength compared to the 0 % ICA group (*P* = 0.000). In addition to statistical significance, the ratio of ICA to printing resin demonstrated an extremely large effect on shear bond strength (partial η^2^ = 0.984), indicating that almost the entire variance in bond strength was attributable to adhesive composition. The type of bond failure is depicted in Fig. 4, Fig. 5. Group 1 (0 % ICA) exhibited predominantly adhesive failures (80 %), which reduced to 60 % for group 2 and to 30 % for Group 3 (50 % ICA). Mixed bond failures were predominant in Group 4 at 50 % respectively, whereas Group 3 had equal predominance of cohesive and mixed failures at 40 % each. A significant positive correlation was observed between shear bond strength and failure mode (Spearman ρ = 0.580, p < 0.001), indicating that specimens with higher bond strength were more likely to exhibit mixed or cohesive failures rather than adhesive failures. A summary table corelating the intergroup analysis of mean shear bond strengths and type of bond failure has been shown in Table 3.

**Table 1:** Groupwise comparison shear bond strength (MPa).

Groups	Mean Shear bond strength ± SD (Mpa)	Median	Interquartile range	95 % confidence interval		p-value
				Lower	Upper	
0 %ICA	1.602 ± 0.140	1.61	0.2	1.5012	1.7028	0.000*
25 % ICA	2.220 ± 0.1271	2.195	0.26	2.129	2.311	
50 % ICA	3.738 ± 0.063	3.715	0.08	3.6929	3.7831	
75 % ICA	3.122 ± 0.0678	3.115	0.12	3.0735	3.1705	
100 % ICA	2.774 ± 0.0721	2.77	0.12	2.7224	2.8256

**Table 2:** Tukey's post hoc multiple comparisons for intergroup comparison of shear bond strength.

Groups		Mean Difference	Std. Error	95 % Confidence Interval		p-value
				Lower	Upper	
0 % ICA	25 % ICA	−.61800*	.04463	−.7448	−.4912	0.000*
	50 %ICA	−2.13600*	.04463	−2.2628	−2.0092	0.000*
	75 %ICA	−1.52000*	.04463	−1.6468	−1.3932	0.000*
	100 % ICA	−1.17200*	.04463	−1.2988	−1.0452	0.000*
25 % ICA	0 % ICA	.61800*	.04463	.4912	.7448	0.000*
	50 %ICA	−1.51800*	.04463	−1.6448	−1.3912	0.000*
	75 %ICA	−.90200*	.04463	−1.0288	−.7752	0.000*
	100 % ICA	−.55400*	.04463	−.6808	−.4272	0.000*
50 %ICA	0 % ICA	2.13600*	.04463	2.0092	2.2628	0.000*
	25 % ICA	1.51800*	.04463	1.3912	1.6448	0.000*
	75 %ICA	.61600*	.04463	.4892	.7428	0.000*
	100 % ICA	.96400*	.04463	.8372	1.0908	0.000*
75 %ICA	0 % ICA	1.52000*	.04463	1.3932	1.6468	0.000*
	25 % ICA	.90200*	.04463	.7752	1.0288	0.000*
	50 %ICA	−.61600*	.04463	−.7428	−.4892	0.000*
	100 % ICA	.34800*	.04463	.2212	.4748	0.000*
100 % ICA	0 % ICA	1.17200*	.04463	1.0452	1.2988	0.000*
	25 % ICA	.55400*	.04463	.4272	.6808	0.000*
	50 %ICA	−.96400*	.04463	−1.0908	−.8372	0.000*
	75 %ICA	−.34800*	.04463	−.4748	−.2212	0.000*

**Table 3:** Summary table corelating the intergroup analysis of mean shear bond strengths and type of bond failure.

Groups	Mean Shear bond strength±SD (Mpa)	Bond failure		
		Adhesive	Cohesive	Mixed
0 %ICA	1.602 ± 0.140	8	0	2
25 % ICA	2.220 ± 0.1271	6	1	3
50 % ICA	3.738 ± 0.063	2	4	4
75 % ICA	3.122 ± 0.0678	3	2	5
100 % ICA	2.774 ± 0.0721	5	1	4

## 4 Discussion

Conventionally, the same photopolymerizing printing resin has been used as an adhesive to bond the 3D-printed denture base and 3D-printed teeth together, despite showing inferior mechanical properties.^20^ The results of this study highlight the potential of ICA:3D-printing resin blend as an effective bonding agent for 3D-printed dental prostheses. Data analysis revealed a significant difference in the shear bond strengths between 3D-printed denture base and tooth samples with the highest shear bond strength achieved by the 50:50 isoamyl cyanoacrylate-resin blend; hence, the null hypothesis was rejected. The extremely large partial eta-squared value (ηp^2^ = 0.984) indicates that the adhesive formulation accounted for almost all the variance in shear bond strength, reinforcing that bond performance in digitally fabricated dentures is overwhelmingly governed by the ICA–resin ratio rather than random variation or secondary factors.

The data revealed a clear influence of the ICA–resin ratio on bonding efficacy, with the 50:50 mixture providing highest adhesion. The 0 % ICA group relied solely on the chemical similarity of the printing resin with the samples, which, although biocompatible and esthetic, lacks strong intrinsic bonding to the relatively inert 3D-printed samples. The performance of this group underscores the inherent limitations of conventional light-cure resin systems when used alone as bonding agents between printed components. They showed a predominant adhesive failure mode. This finding confirms that the interaction between cured denture base resin and tooth resin is insufficient to resist functional stresses, leading to separation at the bonding interface. Group 2 (25 % ICA) suggested that incorporation of ICA likely enhanced adhesion between the samples through both physical and chemical bonding pathways. However, at this concentration, the adhesive may not fully penetrate or co-polymerize with the substrate, limiting its effectiveness. The 25 % ICA group showed a slight improvement, with a shift toward cohesive and mixed failures, indicating an incremental enhancement in the interfacial interaction due to the cyanoacrylate component. Group 3 (50 % ICA) exhibited superior performance, attributed to partial compatibility between the two chemical compounds. The synergistic polymerizations of the polar cyanoacrylate molecules and the photopolymerizing printing resin form an interpenetrating polymer network that strengthens mechanical interlocking and interfacial bonding.^18^ The presence of cohesive failures implied that the internal strength of the adhesive surpassed the strength of the bond interface, a hallmark of strong, durable adhesive bonding. Mixed failures, combining features of both adhesive and cohesive failures, further reflect a well-integrated interface capable of distributing stress more evenly. Upon increasing ICA beyond 50 %, there was a decrease in mean shear bond strength, demonstrating that 50 % ICA provided a critical threshold for forming an interpenetrating polymer network (IPN) between the monomers of the cyanoacrylate and the methacrylate-based components of the 3D printing resin. Group 4 (75 % ICA) showed a decline in bond strength (3.122 ± 0.678 MPa) despite containing more ICA. This suggests that excessive ICA may hinder uniform polymerization during curing and causing brittleness, thereby, leading to a weaker bond. Furthermore, the reduced proportion of light-cure resin at this ratio could limit the formation of a combined strong network across the bonding interface. It is also noteworthy that this group showed comparatively higher variability in shear bond strength, as reflected by its larger standard deviation. This is suggestive of inconsistent bonding performance at this ratio, which may be attributed to difficulties in achieving homogeneous mixing, variability in polymerization kinetics due to the lower proportion of light-cure resin or the antagonistic interaction between the cyanoacrylate and resin compounds. Group 5 (100 % ICA) further confirmed the trend, showing a lower bond strength (2.774 ± 0.072 MPa) than Groups 3 and 4. This indicates that while ICA alone is a strong adhesive in many clinical settings, its bonding to 3D-printed denture materials is suboptimal without co-polymerization with resin-based components. For groups 4 and 5, a greater proportion of mixed failures persisted, but adhesive failures also became more frequent again. This suggests that an excess of ICA may have introduced issues such as decreased mechanical properties, since it tends to form a brittle polymer matrix upon curing, which may compromise its ability to withstand functional stresses at the denture base-tooth interface. The absence of light-cure resin limits copolymerization and the formation of an interpenetrating polymer network, which was likely crucial for the superior performance of the 50:50 blend. Also, ICA alone may exhibit reduced adaptability and wetting on the relatively hydrophobic resin surfaces of the printed denture base and teeth, due to chemical dissimilarity, leading to incomplete interfacial contact that would substantially reduce the surface used for bonding. Together, these factors contributed to the lower bonding performance of pure ICA compared to hybrid formulations. Cohesive failures were less frequent in these higher ICA groups, reflecting a reduction in internal integrity or mechanical compatibility at the bonding interface, which can compromise long-term mechanical performance under masticatory stress. Spearman correlation showed a positive association between shear bond strength and cohesive/mixed failure patterns which further supports the assertion that superior adhesive performance not only increases load-bearing capacity but also shifts the fracture behavior internally towards mixed or cohesive failures, reflecting a more robust and integrated bond at the denture base-tooth interface.

These findings align with previous research on cyanoacrylate adhesives in biomedical applications.^15^^,^^17^^,^^22^ They are clinical studies that have shown that cyanoacrylate adhesives can be a viable alternative to sutures in different surgical procedures. Another study reported that ICA achieved the highest shear bond strength among cyanoacrylates for 3D-printed dentures. Unlike their study, which tested pure cyanoacrylate formulations, our work demonstrates that blending ICA with light-cure resin further enhances adhesion, with the 50:50 ratio yielding highest results. By blending with a photopolymerizable resin, the adhesive gains improved adaptability to the surface morphology of the 3D-printed denture base and teeth.^24^ In an in-vitro study comparing the effects of surface treatments, Boonpitak et al. compared conventional resin, sandblasting, heat-cure polymerization and methyl methacrylate liquid conditioning and reported heat-polymerization to achieve the highest shear bond strength followed by sandblasting and liquid conditioning groups.^23^ Pereira et al. evaluated different surface treatments and concluded that only sandblasting was able to roughen the surface enough, that enabled a significant increase in shear bond strength of both non-aged and aged 3D-printed denture samples.^6^ Even though previous studies showed higher shear bond strength values with various parameters, they cannot be compared due to heterogeneous methodologies. Although surface treatments such as sandblasting or chemical conditioning were intentionally excluded to purely evaluate the adhesive's effects, these techniques may act synergistically with the adhesive in clinical scenarios, potentially further improving bond durability. Given the increasing adoption of 3D printing in dentistry, the development of specialized bonding agents tailored for these materials is essential. The challenges associated with bonding 3D-printed denture bases, including differences in surface energy and polymerization shrinkage, highlight the need for continued research in this field.

A major strength of the present study lies in its novelty, as it is the first to systematically evaluate blends of ICA with light-cure 3D printing resin for bonding denture bases and teeth. Previous work has largely focused on surface treatments or conventional adhesives, whereas this study introduces a novel hybrid adhesive concept tailored specifically for the digital denture workflow. In this study, the samples were bonded only with the customized adhesive mixture, without any surface modifications so as to precisely analyze the effect of the cyanoacrylate adhesive ratios, while avoiding any confounding factors caused by the surface modifications. Strict adherence to internationally recognized standards (ISO/TS 19736), which enhanced the reliability, reproducibility, and comparability of the results with the use of a randomized allocation strategy and a systematic experimental design that helped in minimizing bias and ensured consistency across groups. The study's statistical design, including a priori power calculation, ANOVA with post hoc comparisons, and verification of variance homogeneity, adds to its methodological strength. The addition of failure mode analysis alongside quantitative shear testing provided a comprehensive understanding of bond integrity between 3D-printed denture components.

Despite promising results, the study has certain limitations. First, even though standardized, it uses simplified shear tests on flat samples rather than full anatomic denture teeth in actual prosthetic contours, which may not replicate the bonding surface area available clinically in dentures. Second, only a single type of resin was tested which restricts the generalizability of the findings to other material systems. Third, the effect of long-term water storage and fatigue cycling was not evaluated in this study, which could influence the long-term durability under intraoral conditions. No material compatibility tests were performed, which could be a part of future studies, that would validate the chemical rationale for the interaction between cyanoacrylate and 3D-printing resin. Additionally, although the sample size was calculated using G*Power software which was adequate for detecting statistical differences, the relatively small number of specimens per group may limit the detection of subtle effects. The study was conducted at a single centre, and variability in printing parameters, resin polymer composition, polymerization reaction kinetics, post-curing, or adhesive handling across different settings could influence outcomes. Finally, operator handling during adhesive mixing and application may introduce potential bias, despite efforts to standardize the methodological procedures.

While the present findings demonstrate promising results for the 50:50 ICA-resin formulation, they should be regarded as preliminary. From a clinical perspective, optimizing adhesive formulations can significantly improve the longevity and reliability of 3D-printed dentures. Integration of this 50:50 blend in digital denture workflows can be beneficial in reducing the risk of debonding and fracture, thereby decreasing the demand for frequent repairs and improving the longevity of the prostheses.^9^ For patients, this translates into greater confidence, functional efficiency, and overall satisfaction with complete dentures fabricated through digital workflows. However, the study was conducted under controlled in-vitro conditions, and further validation is necessary before clinical recommendations can be made. Specifically, multi-centric, long-term durability assessments such as thermocycling, water aging, and cyclic mechanical loading are required to simulate the intraoral environment and evaluate resistance to degradation over time. Such testing would provide greater insight into clinical applicability and long-term performance of these adhesive formulations. The influence of different photoinitiators, filler content, and surface treatments on bonding efficiency should also be explored. Additionally, the feasibility of commercializing a dual-component adhesive for digital dentures from a manufacturing point of view should be evaluated. Future studies could include FTIR or DSC analysis of the ICA-resin mixture to confirm chemical interactions and polymerization compatibility, durability under cyclic loading, evaluation of multiple bonding systems, enhancing reproducibility of results and then performing clinical trials to validate practical implications.

## 5 Conclusion

This study demonstrates that the shear bond strength of 3D-printed denture bases can be significantly improved by incorporating ICA. Among the tested formulations, the 50:50 ratio of ICA to 3D-printing resin exhibited the highest bond strength, indicating a balance between adhesion and mechanical properties. These findings provide a foundation for further research and potential clinical applications in prosthodontics. Future studies should include thermocycling, fatigue loading, and in vivo validation to confirm long-term performance.

## Ethical clearance

Being an in-vitro study, it did not require Institutional Human Ethical Clearance.

## Sources of funding

There were no sources of funding for this study.

## Declaration of competing interest

The authors declare that they have no known competing financial interests or personal relationships that could have appeared to influence the work reported in this paper.
